# Supplementary material for: Distance effect of single atoms on stability of cobalt oxide catalysts for acidic oxygen evolution
Source: Nat Commun. 2024 Feb 26;15:1767. doi: 10.1038/s41467-024-46176-0 (PMC10897172; doi:10.1038/s41467-024-46176-0)
Supplement: Supplementary file 1 — Supplementary Information [file 41467_2024_46176_MOESM1_ESM.pdf]

Supplementary Information for

Distance effect of single atoms on stability of cobalt oxide catalysts for acidic oxygen evolution

Zhirong Zhang[1†], Chuanyi Jia[2†], Peiyu Ma[3†], Chen Feng[1], Jin Yang[1], Junming Huang[1], Jiana Zheng[1], Ming Zuo[1], Mingkai Liu[4], Shiming Zhou[1]*, Jie Zeng[1,4]*

[1]Hefei National Research Center for Physical Sciences at the Microscale, Key Laboratory of Strongly-Coupled Quantum Matter Physics of Chinese Academy of Sciences, Key Laboratory of Surface and Interface Chemistry and Energy Catalysis of Anhui Higher Education Institutes, Department of Chemical Physics, University of Science and Technology of China, Hefei, Anhui 230026, P. R. China

[2]Guizhou Provincial Key Laboratory of Computational Nano-Material Science, Institute of Applied Physics, Guizhou Education University, Guiyang, Guizhou 550018, P. R. China

[3]National Synchrotron Radiation Laboratory, Key Laboratory of Precision and Intelligent Chemistry, *i*ChEM (Collaborative Innovation Center of Chemistry for Energy Materials), University of Science and Technology of China, Hefei, Anhui 230026, P. R. China

[4]School of Chemistry & Chemical Engineering, Anhui University of Technology, Ma'anshan, Anhui 243002, P. R. China

*Corresponding author E-mail: zhousm@ustc.edu.cn (S.Z.); zengj@ustc.edu.cn (J.Z.)

[†]These authors contributed equally to this work.

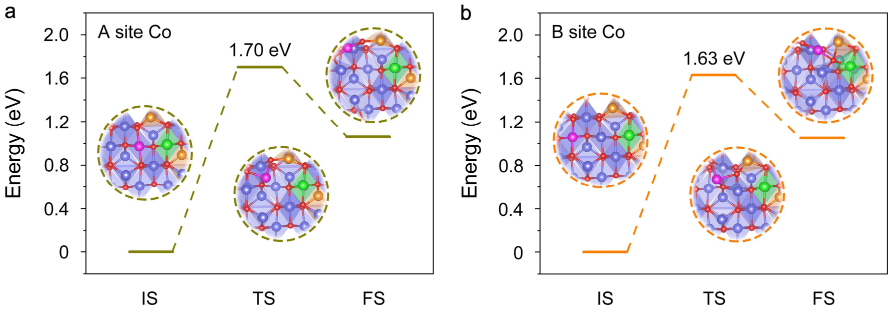

Supplementary Figure 1 | Calculated migration energies of Co atoms on $Ir_1/Cu_{0.3}Co_{2.7}O_4$. a, b, Calculated migration energies of the nearest (A site) (**a**) and next nearest (B site) (**b**) Co atom to the Ir single atom. The inset structures represent the initial state (IS), transition state (TS), and final state (FS), respectively. Red, blue, brown, and green spheres represent O, Co, Cu, and Ir atoms, respectively. Pink spheres represent migrated Co atoms.

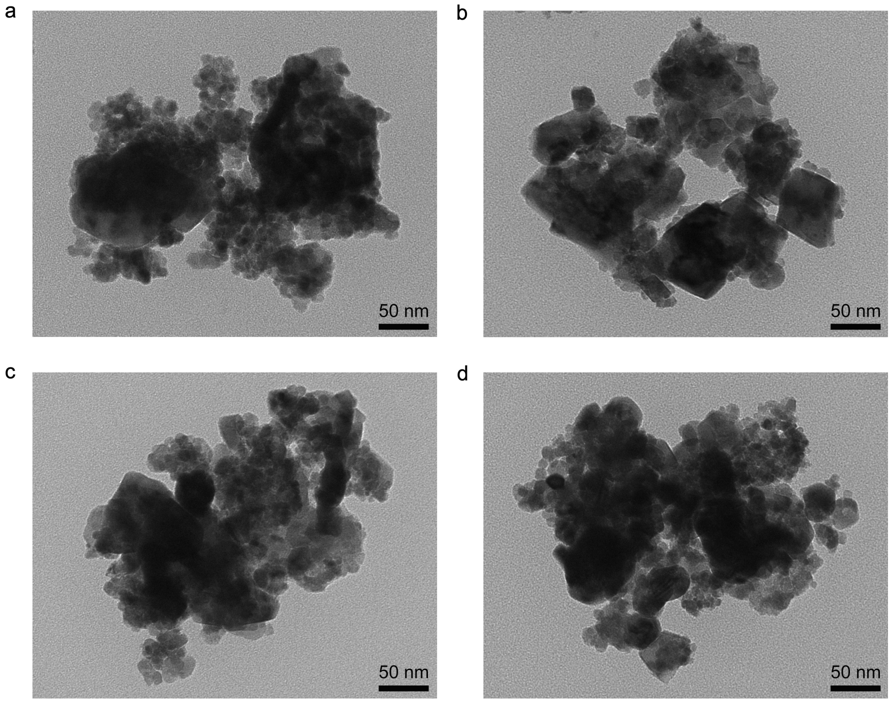

Supplementary Figure 2 | Morphological characterizations. a-d, TEM images of $Cu_{0.3}Co_{2.7}O_4$ (**a**), $Ir_1/Cu_{0.3}Co_{2.7}O_4$ with d = 1.1 nm (**b**), $Ir_1/Cu_{0.3}Co_{2.7}O_4$ with d = 0.8 nm (**c**), and $Ir_1/Cu_{0.3}Co_{2.7}O_4$ with d = 0.6 nm (**d**).

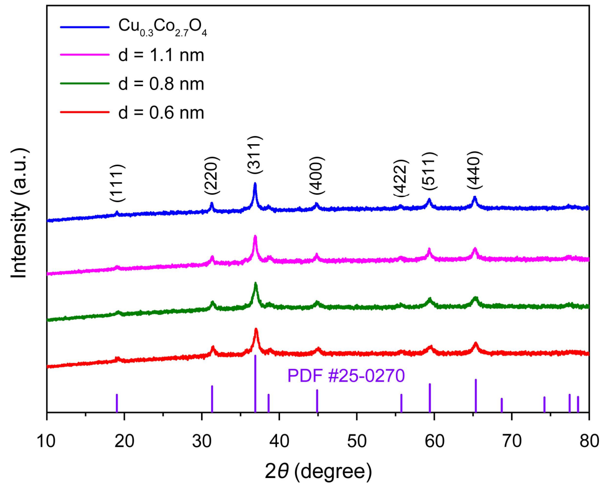

Supplementary Figure 3 | Structure characterization. XRD patterns of $Cu_{0.3}Co_{2.7}O_4$ and $Ir_1/Cu_{0.3}Co_{2.7}O_4$ with different Ir-Ir distances.

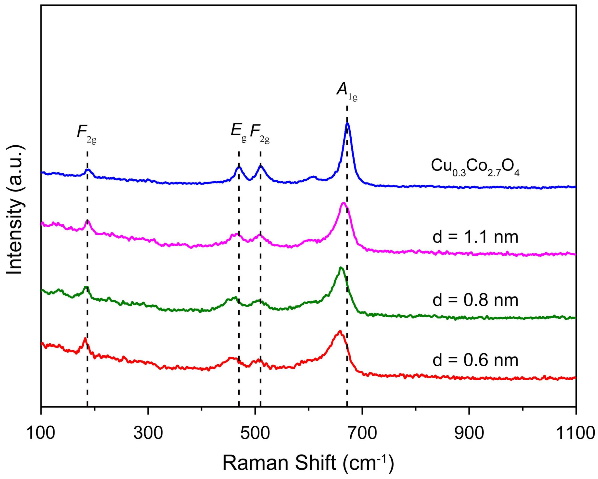

Supplementary Figure 4 | Raman characterizations. Raman spectra of $Cu_{0.3}Co_{2.7}O_4$ and $Ir_1/Cu_{0.3}Co_{2.7}O_4$ with different Ir-Ir distances.

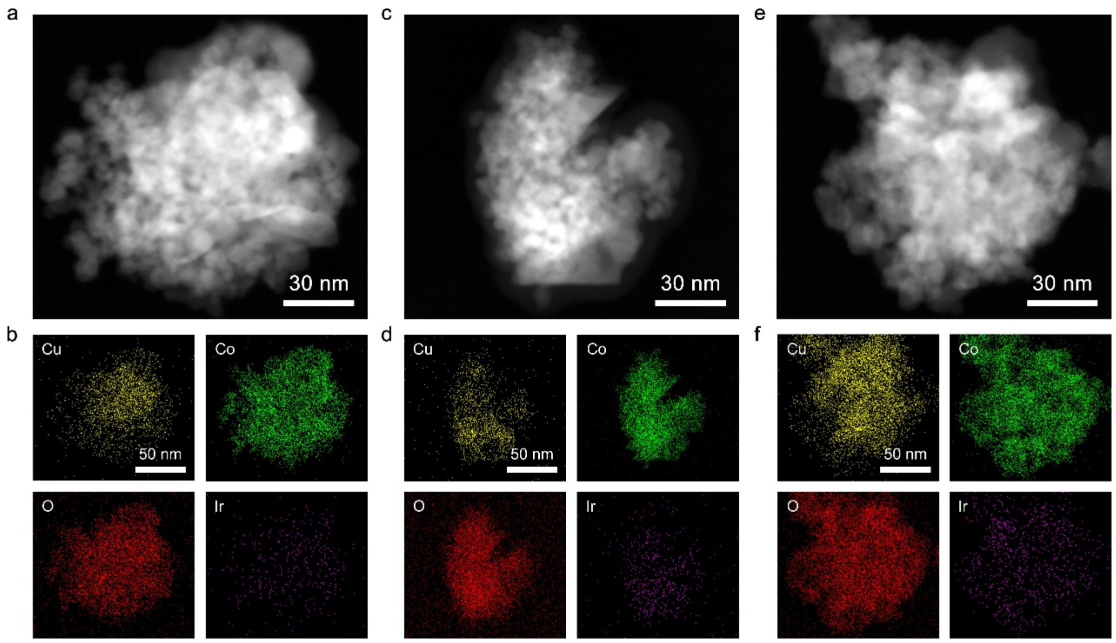

Supplementary Figure 5 | Morphological characterizations and elemental distributions of $Ir_1/Cu_{0.3}Co_{2.7}O_4$ with different Ir-Ir distances. a, **c**, **e**, TEM images of $Ir_1/Cu_{0.3}Co_{2.7}O_4$ with d = 1.1 nm (**a**), 0.8 nm (**c**), and 0.6 nm (**e**). **b**, **d**, **f**, EDX elemental mapping of $Ir_1/Cu_{0.3}Co_{2.7}O_4$ with d = 1.1 nm (**b**), 0.8 nm (**d**), and 0.6 nm (**f**).

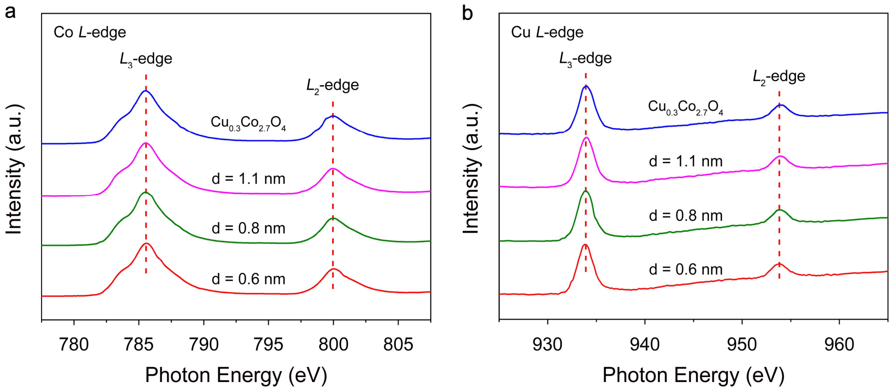

Supplementary Figure 6 | Electronic structure characterizations. a, Co *L*-edge XAS spectra of $Cu_{0.3}Co_{2.7}O_4$ and $Ir_1/Cu_{0.3}Co_{2.7}O_4$ with different Ir-Ir distances. **b**, Cu *L*-edge XAS spectra of $Cu_{0.3}Co_{2.7}O_4$ and $Ir_1/Cu_{0.3}Co_{2.7}O_4$ with different Ir-Ir distances.

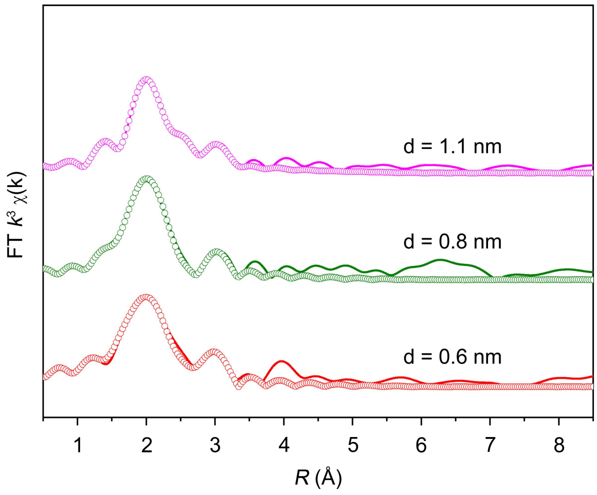

Supplementary Figure 7 | EXAFS fitting results. Experimental and fitting EXAFS spectra at the Ir L_3-edge of $Ir_1/Cu_{0.3}Co_{2.7}O_4$ with different Ir-Ir distances. The experimental and fitting results are indicated as solid and circles lines, respectively.

Supplementary Table 1. Fitting results of Ir L_3-edge EXAFS spectra for $Ir_1/Cu_{0.3}Co_{2.7}O_4$ with different Ir-Ir distances.

Samples	Path	R (Å)	CNs	σ^2 (10^{-3})	ΔE_0 (eV)	R-factor
d = 1.1 nm	Ir-O	1.99	5.8	8.5	8.8	0.010
	Ir-O-Co	3.02	2.8	7.4	-8.5	
d = 0.8 nm	Ir-O	1.99	6.3	6.5	8.8	0.012
	Ir-O-Co	3.05	2.8	8.8	-7.1	
d = 0.6 nm	Ir-O	1.99	6.3	8.0	9.0	0.010
	Ir-O-Co	3.03	3.0	6.0	-8.8	

R, distance between absorber and backscatter atoms; CNs, coordination number; σ^2, Debye-Waller factors; ΔE_0, inner potential correction that accounts for the difference in the inner potential between the sample and the references. R-factor: goodness of fit.

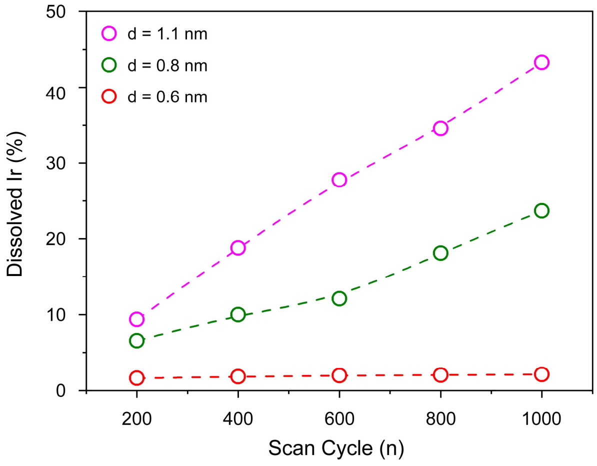

Supplementary Figure 8 | Stability evaluation for $Ir_1/Cu_{0.3}Co_{2.7}O_4$ with d = 1.1, 0.8, and 0.6 nm. Dissolution of Ir species under different scan cycles of $Ir_1/Cu_{0.3}Co_{2.7}O_4$ with different Ir-Ir distances.

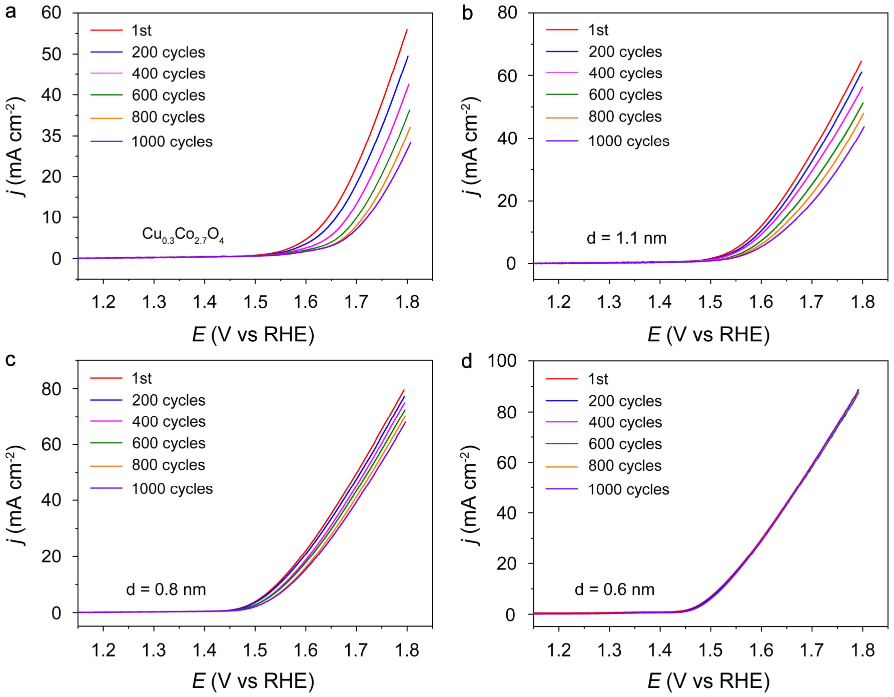

Supplementary Figure 9 | Stability evaluation towards acidic oxygen evolution on Ti felt. a-d, Polarization curves of $Cu_{0.3}Co_{2.7}O_4$ (**a**) and $Ir_1/Cu_{0.3}Co_{2.7}O_4$ with d = 1.1 nm (**b**), d = 0.8 nm (**c**), and d = 0.6 nm (**d**) at different scan cycles in 0.1 M $HClO_4$ electrolyte. The displayed polarization curves are the 1, 200, 400, 600, 800, and 1000 cycles, respectively. No activated carbon was added at the electrode preparation stage. The ohmic electrolyte resistance of catalysts on Ti felt was measured to be 0.5 Ω.

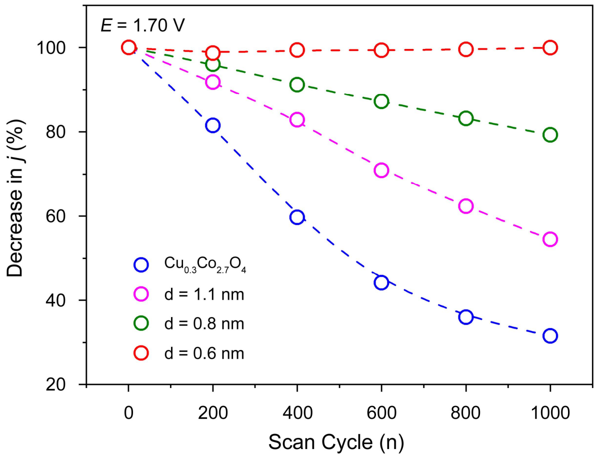

Supplementary Figure 10 | Decrease in current densities at different scan cycles of $Cu_{0.3}Co_{2.7}O_4$ and $Ir_1/Cu_{0.3}Co_{2.7}O_4$ with different Ir-Ir distances on Ti felt. The selected overpotential was 1.70 V (*E* vs RHE) for all catalysts.

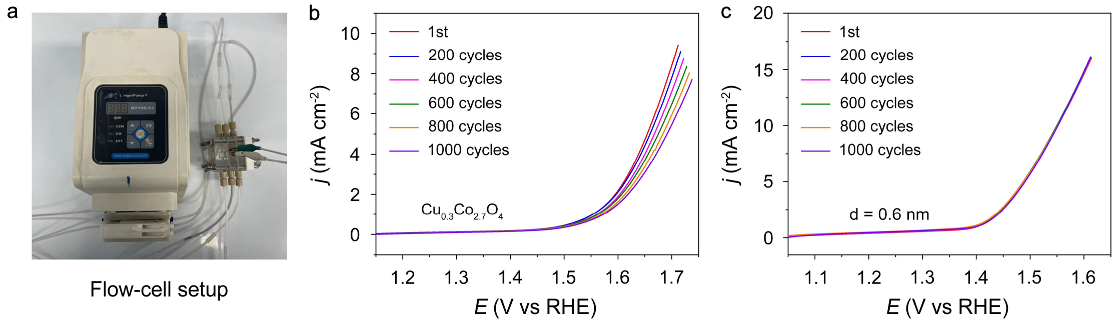

Supplementary Figure 11 | Stability evaluation of $Ir_1/Cu_{0.3}Co_{2.7}O_4$ with d = 0.6 nm for acidic OER in a flow-cell setup. **a**, Optical image of the flow-cell setup. **b**, **c**, Polarization curves of $Cu_{0.3}Co_{2.7}O_4$ (**b**) and $Ir_1/Cu_{0.3}Co_{2.7}O_4$ with d = 0.6 nm (**c**) at different scan cycles in 0.1 M $HClO_4$ electrolyte. The displayed polarization curves are the 1, 200, 400, 600, 800, and 1000 cycles, respectively. The ohmic electrolyte resistance was measured to be 15 Ω.

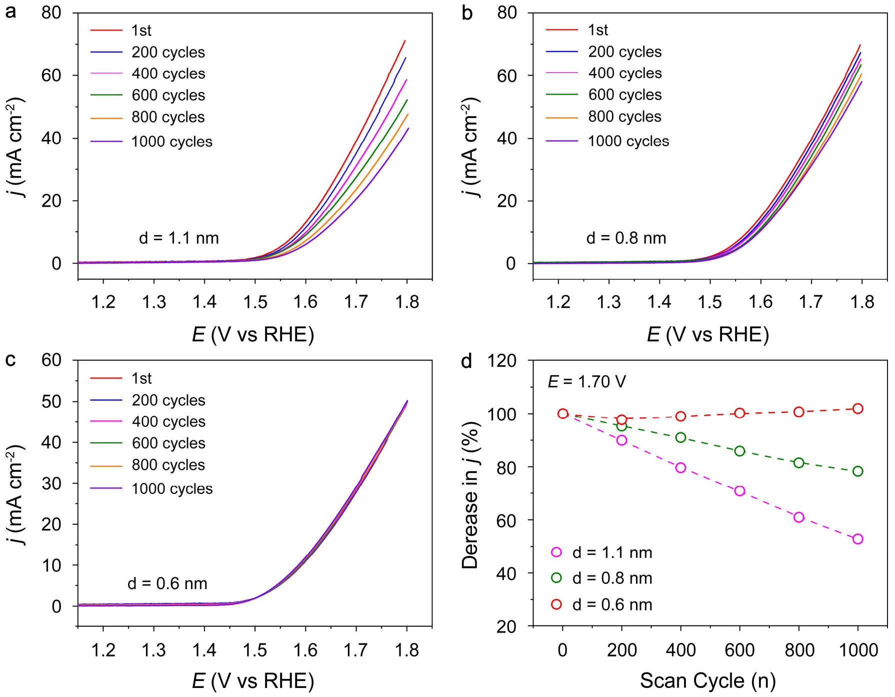

Supplementary Figure 12 | Stability evaluation towards acidic oxygen evolution on Ti felt. The mass loadings of Ir species on the electrode were fixed at 25 μg cm^{-2}. a-c, Polarization curves of $Ir_1/Cu_{0.3}Co_{2.7}O_4$ with d = 1.1 nm (**a**), d = 0.8 nm (**b**), and d = 0.6 nm (**c**) at different scan cycles in 0.1 M $HClO_4$ electrolyte. The displayed polarization curves are the 1, 200, 400, 600, 800, and 1000 cycles, respectively. No activated carbon was added at the catalyst preparation stage. **d**, Decrease in current densities under different scan cycles of $Ir_1/Cu_{0.3}Co_{2.7}O_4$ with d = 1.1 nm, 0.8 nm, and 0.6 nm containing similar loadings of Ir species. The selected overpotential was 1.70 V (*E* vs RHE) for all catalysts. The ohmic electrolyte resistance of catalysts on Ti felt was measured to be 0.5 Ω.

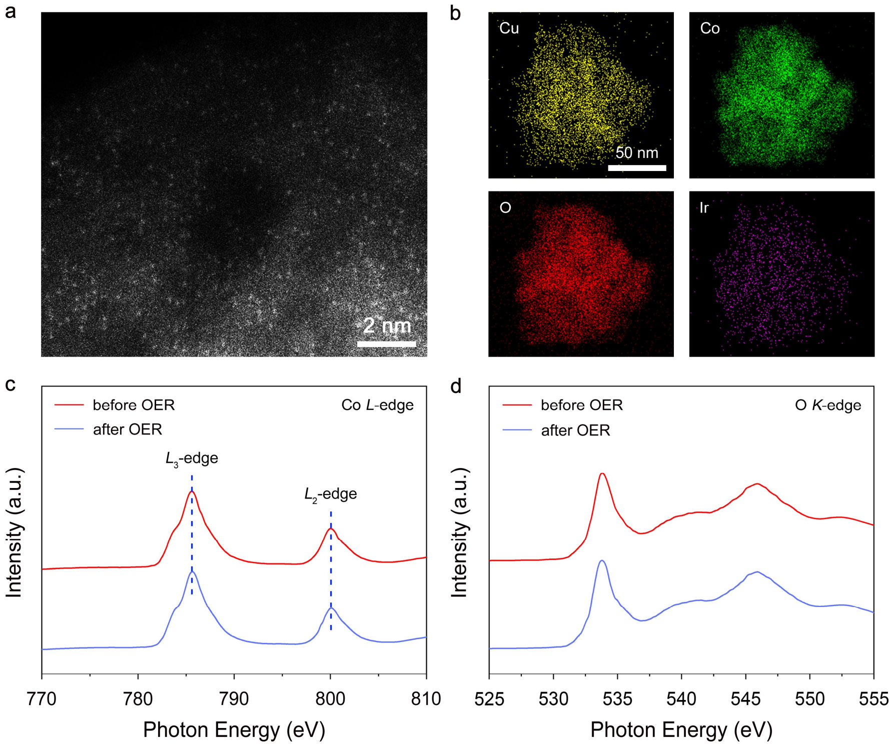

Supplementary Figure 13 | Structural characterizations of $Ir_1/Cu_{0.3}Co_{2.7}O_4$ with d = 0.6 nm after acidic OER. a, HAADF-STEM image of $Ir_1/Cu_{0.3}Co_{2.7}O_4$ with d = 0.6 nm after acidic OER. **b**, EDX elemental mapping of $Ir_1/Cu_{0.3}Co_{2.7}O_4$ with d = 0.6 nm after acidic OER. **c**, **d**, Co *L*-edge (**c**) and O *K*-edge (**d**) XAS spectra of $Ir_1/Cu_{0.3}Co_{2.7}O_4$ with d = 0.6 nm after acidic OER. Co *L*-edge and O *K*-edge XAS spectra of $Ir_1/Cu_{0.3}Co_{2.7}O_4$ before acidic OER were used as a reference.

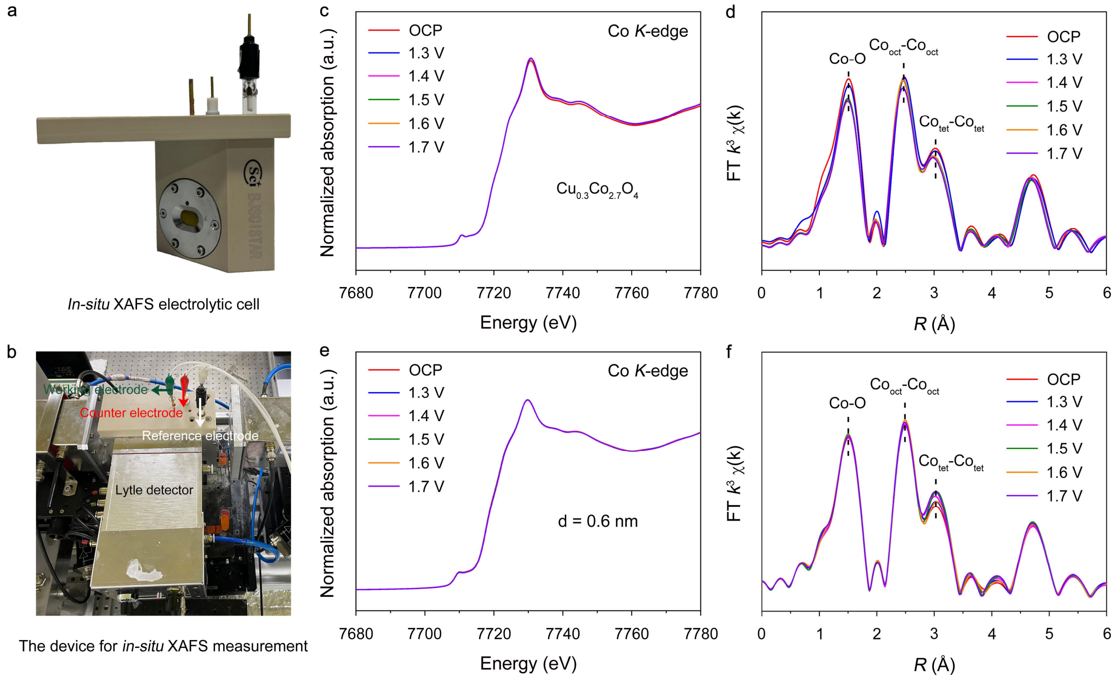

Supplementary Figure 14 | *In-situ* spectroscopic characterizations. **a**, **b**, Optical images of the *in-situ* XAFS electrolytic cell (**a**) and the device for *in-situ* XAFS measurement (**b**). **c**, **d**, *In-situ* Co *K*-edge XANES (**c**) and EXAFS (**d**) spectra of $Cu_{0.3}Co_{2.7}O_4$ at different applied potentials. **e**, **f**, *In-situ* Co *K*-edge XANES (**e**) and EXAFS (**f**) spectra of $Ir_1/Cu_{0.3}Co_{2.7}O_4$ with d = 0.6 nm at different applied potentials.

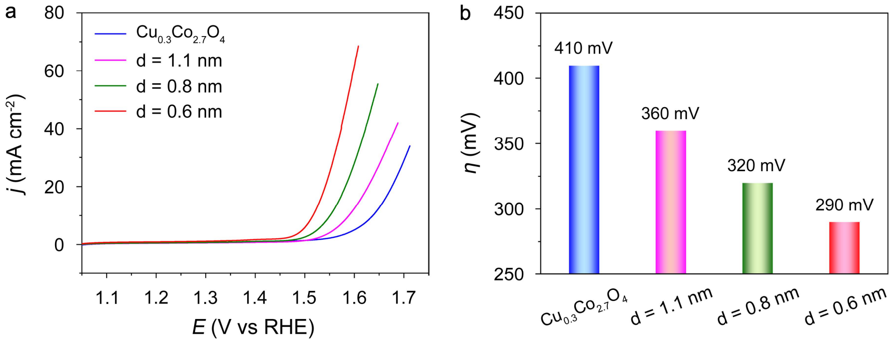

Supplementary Figure 15 | Electrocatalytic characterizations. **a**, Polarization curves of $Cu_{0.3}Co_{2.7}O_4$ and $Ir_1/Cu_{0.3}Co_{2.7}O_4$ with d = 1.1, 0.8, and 0.6 nm. **b**, Overpotentials of $Cu_{0.3}Co_{2.7}O_4$ and $Ir_1/Cu_{0.3}Co_{2.7}O_4$ with d = 1.1, 0.8, and 0.6 nm at a current density of 10 mA cm^{-2}.

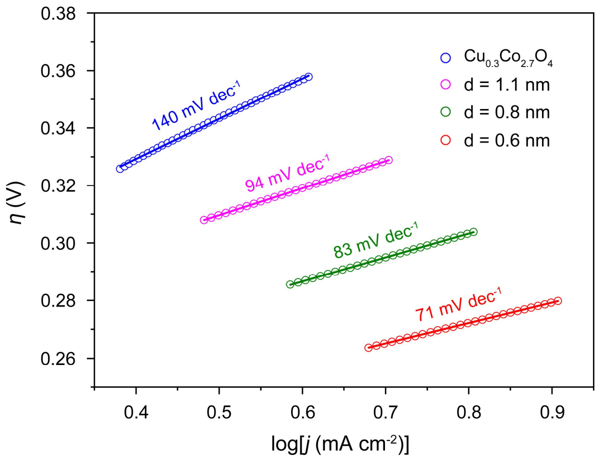

Supplementary Figure 16 | Tafel slopes. Tafel slopes of $Cu_{0.3}Co_{2.7}O_4$ and $Ir_1/Cu_{0.3}Co_{2.7}O_4$ with different Ir-Ir distances.

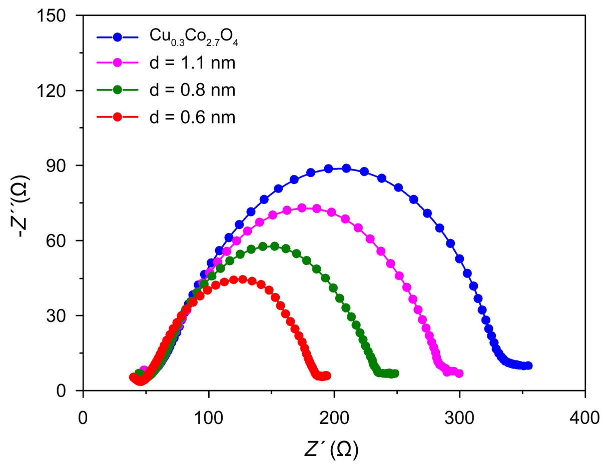

Supplementary Figure 17 | Electrochemical impedance spectra. Electrochemical impedance spectra of $Cu_{0.3}Co_{2.7}O_4$ and $Ir_1/Cu_{0.3}Co_{2.7}O_4$ with different Ir-Ir distances.

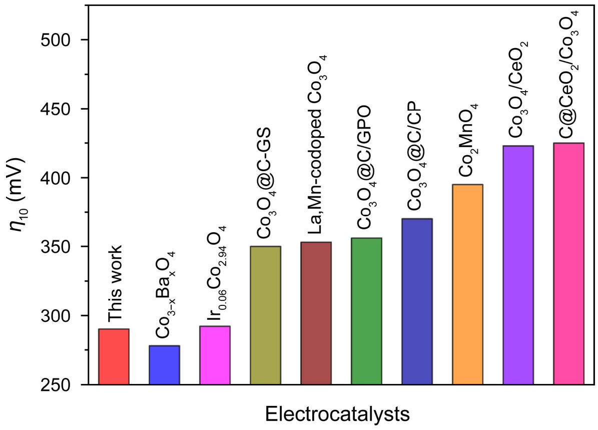

Supplementary Figure 18 | Electrocatalytic performance comparison. Comparison of overpotentials at a current density of 10 mA cm^{-2} for currently reported acidic OER catalysts.

Supplementary Table 2. Comparison of oxygen evolution performance for recently reported Co-based catalysts in acidic electrolyte.

Catalysts	Electrolyte	Overpotential (mV) @ j = 10 mA cm^{-2}	Ref.
**$Ir_1/Cu_{0.3}Co_{2.7}O_4$ with d = 0.6 nm**	**0.1 M $HClO_4$**	**290**	**This work**
$Co_{3-x}Ba_xO_4$	0.5 M H_2SO_4	278	R1
$Ir_{0.06}Co_{2.94}O_4$	1.0 M $HClO_4$	292	R2
Co_3O_4@C-GS	pH 1.0 H_2SO	350	R3
La,Mn-codoped Co_3O_4	0.1 M $HClO_4$	353	R4
Co_3O_4@C/GPO	1.0 M H_2SO_4	356	R5
Co_3O_4@C/CP	0.5 M H_2SO_4	370	R6
Co_2MnO_4	pH 1.0 H_2SO_4	395	R7
Co_3O_4/CeO_2	0.5 M H_2SO_4	423	R8
C@CeO_2/Co_3O_4	0.5 M H_2SO_4	425	R9

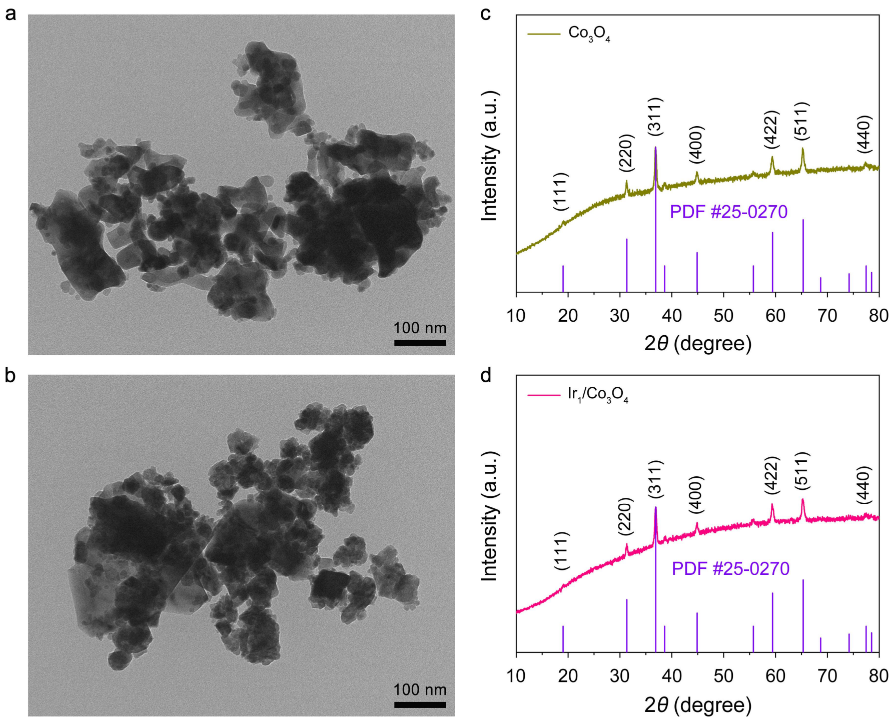

Supplementary Figure 19 | Morphological characterizations and XRD patterns of Co_3O_4 and Ir_1/Co_3O_4. a, **b**, TEM images of Co_3O_4 (**a**) and Ir_1/Co_3O_4 (**b**). **c**, **d**, XRD patterns of Co_3O_4 (**c**) and Ir_1/Co_3O_4 (**d**).

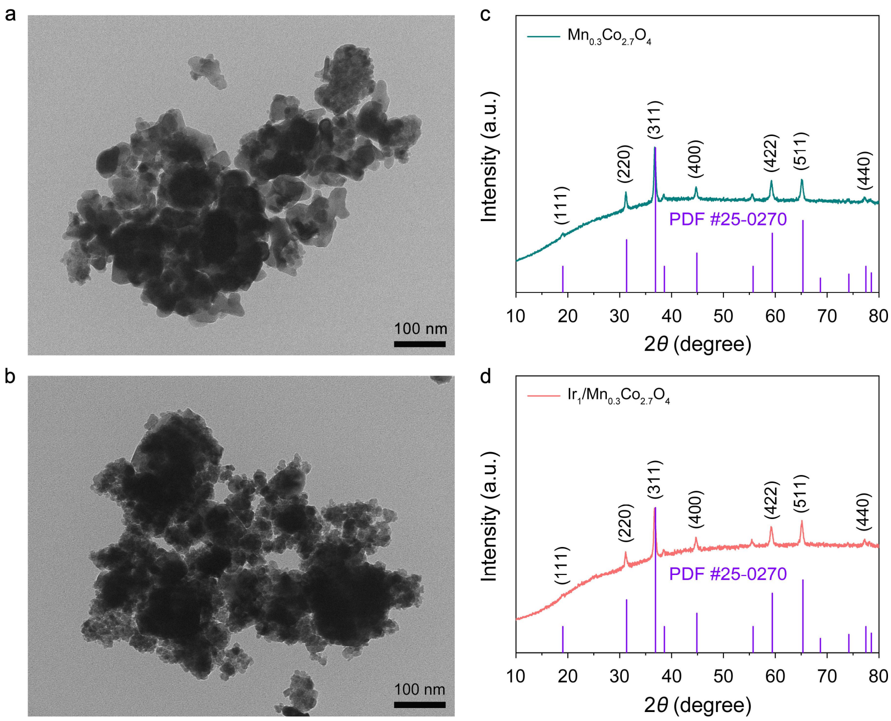

Supplementary Figure 20 | Morphological characterizations and XRD patterns of $Mn_{0.3}Co_{2.7}O_4$ and $Ir_1/Mn_{0.3}Co_{2.7}O_4$. a, **b**, TEM images of $Mn_{0.3}Co_{2.7}O_4$ (**a**) and $Ir_1/Mn_{0.3}Co_{2.7}O_4$ (**b**). **c**, **d**, XRD patterns of $Mn_{0.3}Co_{2.7}O_4$ (**c**) and $Ir_1/Mn_{0.3}Co_{2.7}O_4$ (**d**).

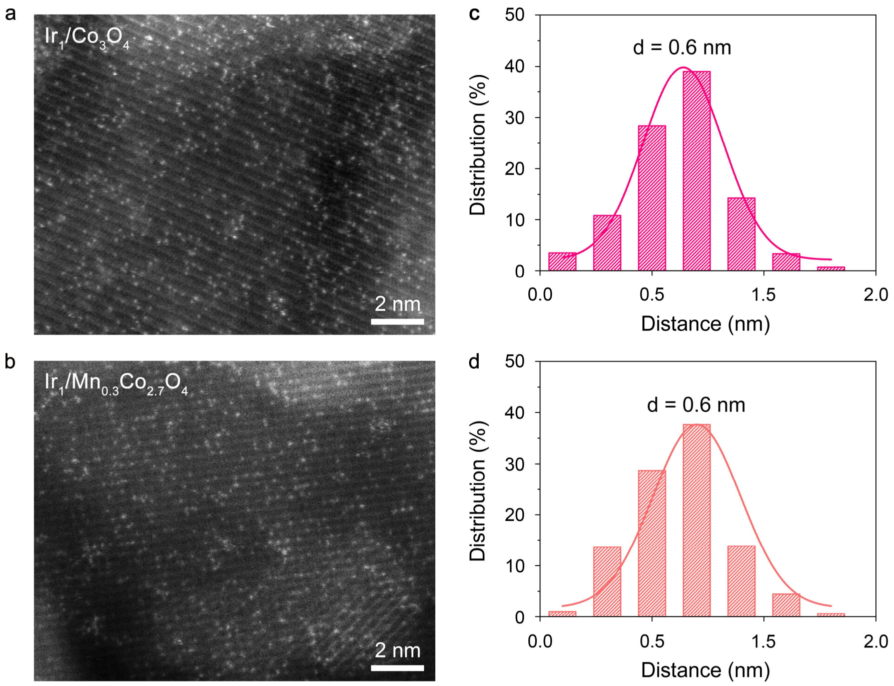

Supplementary Figure 21 | Structure characterizations of Ir_1/Co_3O_4 and $Ir_1/Mn_{0.3}Co_{2.7}O_4$. a, b, HAADF-STEM image of Ir_1/Co_3O_4 (**a**) and $Ir_1/Mn_{0.3}Co_{2.7}O_4$ (**b**). **c**, **d**, Distance distribution of Ir single atoms in the HAADF-STEM images of Ir_1/Co_3O_4 (**c**) and $Ir_1/Mn_{0.3}Co_{2.7}O_4$ (**d**).

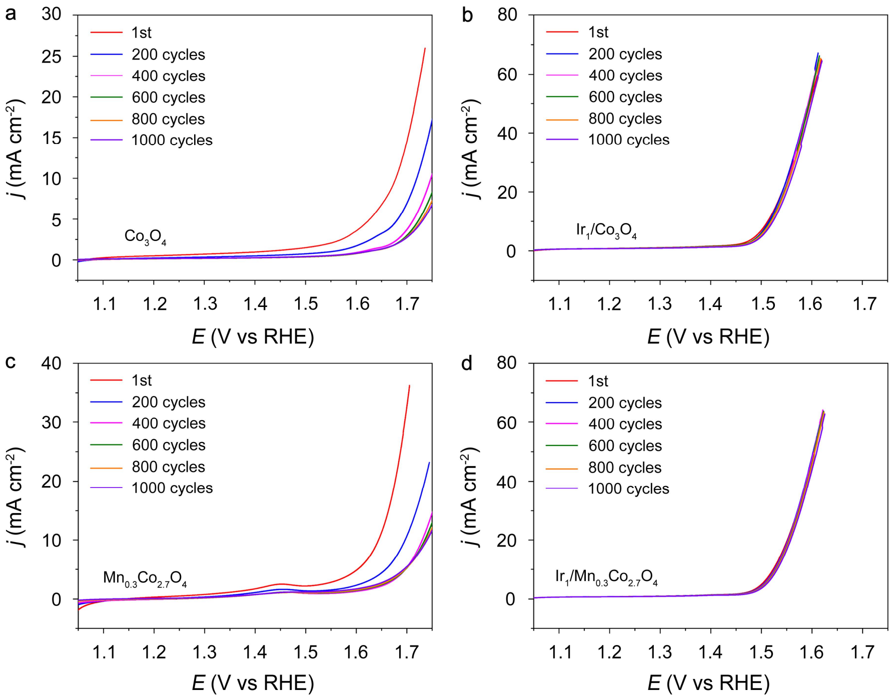

Supplementary Figure 22 | Electrocatalytic evaluation towards acidic oxygen evolution. a-d, Polarization curves of Co_3O_4 (**a**), Ir_1/Co_3O_4 (**b**), $Mn_{0.3}Co_{2.7}O_4$ (**c**), and $Ir_1/Mn_{0.3}Co_{2.7}O_4$ (**d**) under different scan cycles. The measurements were conducted in 0.1 M $HClO_4$.

Supplementary References

1. Wang, N. et al. Doping shortens the metal/metal distance and promotes OH coverage in non-noble acidic oxygen evolution reaction catalysts. *J. Am. Chem. Soc.* **145**, 7829-7836 (2023).
2. Shan, J. et al. Short-range ordered iridium single atoms integrated into cobalt oxide spinel structure for highly efficient electrocatalytic water oxidation. *J. Am. Chem. Soc.* **143**, 5201-5211 (2021).
3. Liu, Z. et al. Interface engineering a high content of Co^{3+} sites on Co_3O_4 nanoparticles to boost acidic oxygen evolution. *Langmuir* **39**, 16415-16421 (2023).
4. Chong, L. et al. La- and Mn-doped cobalt spinel oxygen evolution catalyst for proton exchange membrane electrolysis. *Science* **380**, ade1499 (2023).
5. Yu, J. et al. Sustainable oxygen evolution electrocatalysis in aqueous 1 M H_2SO_4 with earth abundant nanostructured Co_3O_4. *Nat. Commun.* **13**, 4341 (2022).
6. Yang, X. et al. Highly acid-durable carbon coated Co_3O_4 nanoarrays as efficient oxygen evolution electrocatalysts. *Nano Energy* **25**, 42-50 (2016).
7. Li, A. et al. Enhancing the stability of cobalt spinel oxide towards sustainable oxygen evolution in acid. *Nat. Catal.* **5**, 109-118 (2022).
8. Huang, J. et al. Modifying redox properties and local bonding of Co_3O_4 by CeO_2 enhances oxygen evolution catalysis in acid. *Nat. Commun.* **12**, 3036 (2021).
9. Liu, H. et al. Boosting CeO_2/Co_3O_4 heterojunctions acidic oxygen evolution via promoting OH coverage. *ACS Appl. Energy Mater.* **6**, 8949-8956 (2023).
